# Basic Anthropometric Measures in Acute Myocardial Infarction Patients and Individually Sex- and Age-Matched Controls from the General Population

**DOI:** 10.1155/2018/3839482

**Published:** 2018-10-02

**Authors:** Göran Nilsson, Pär Hedberg, Jerzy Leppert, John Ohrvik

**Affiliations:** ^1^Center for Clinical Research, Region Vastmanland-Uppsala University, Hospital of Vastmanland, Västerås, Sweden; ^2^Department of Clinical Physiology, Hospital of Vastmanland, Västerås, Sweden

## Abstract

We compared weight, height, waist and hip circumferences (hip), body mass index (BMI), and waist-to-hip ratio in acute myocardial infarction (MI) patients and individually sex- and age-matched control subjects from the general population in the catchment area of the patients and predicted the risk of MI status by these basic anthropometric measures. The study cohort comprised 748 patients ≤80 years of age with acute MI from a major Swedish cardiac center and their individually sex- and age-matched controls. The analyses were stratified for sex and age (≤65/≥66 years). Risk of MI was assessed by conditional logistic regression. A narrow hip in men ≥66 years was the single strongest risk factor of MI among the anthropometric measures. The combination of *hip and weight* was particularly efficient in discriminating men ≥66 years with MI from their controls (area under the receiver operating characteristic (AUROC) curve = 0.82). In men ≤65 years, the best combination was hip, BMI, and height (AUROC = 0.79). In women ≥66 years, the best discriminatory model contained only waist-to-hip ratio (AUROC = 0.67), whereas in women ≤65 years, the best combination was hip and BMI (AUROC = 0.68). A narrow hip reasonably reflects small gluteal muscles. This finding might suggest an association between MI and sarcopenia, possibly related to deficiencies in physical activity and nutrition.

## 1. Introduction

Four anthropometric measures are commonly registered in the health care: weight, height, waist circumference (waist), and hip circumference (hip). Additionally, two quotients derived from these measures, body mass index (BMI, weight kg/height^2^ m^2^) and waist-to-hip ratio (waist/hip), are often used.

Case-control studies compare phenotypes of cases and their controls determined approximately at the time of the index event. This implies a fundamental difference from prospective follow-up studies that register phenotypes of future cases and noncases at a considerable time, usually several years, before the event. Anthropometric measures may change significantly during these years. However, in contrast to many biochemical variables, for instance blood glucose, the anthropometric variables are probably not affected by the acute myocardial infarction (MI) event. Thus, the anthropometric measures in acute MI patients are reasonably valid at least for a few months before the MI event.

Prospective studies have demonstrated an association between future coronary events and high BMI and weight gain [1, 2] as well as large waist and waist/hip [3, 4].

Contrarily to prospective follow-up studies, there are few case-control studies of anthropometric measures involving MI patients. Yusuf et al. [5] reported from a worldwide case-control study of acute MI patients using mainly attendants or relatives of patients from noncardiac wards. They found that BMI and waist/hip were larger in patients than in controls. Likewise, Kahn et al. [6], Azevedo et al. [7], and Oliveira et al. [8] reported a larger waist/hip in MI patients than in nonmatched randomly selected control subjects from the general population of the catchment area of the patients.

The objective of this study was to explore the associations between basic anthropometric phenotypes and MI status in a sex- and age-matched case-control population.

## 2. Methods

### 2.1. Study Design

**Figure 1:**
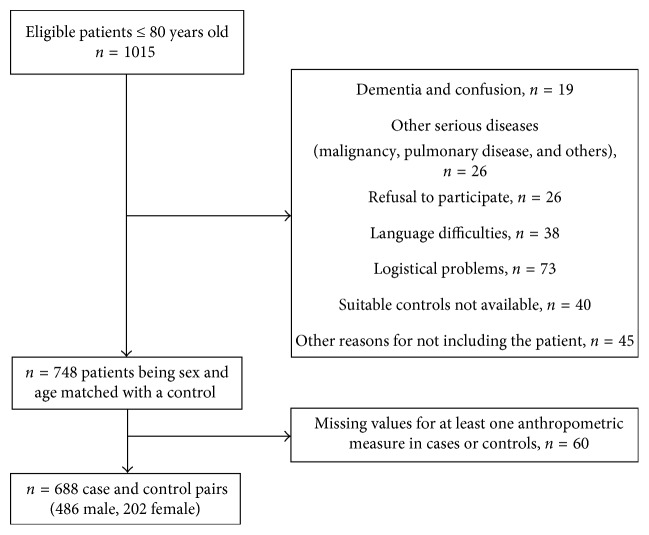
Flow chart of the myocardial infarction patients and their matched controls.

The Vastmanland Myocardial Infarction Study (VaMIS) recruited consecutive acute MI patients admitted to the coronary care unit of the Hospital of Vastmanland, Vasteras, Sweden, between November 2005 and May 2011; https://clinicaltrials.gov/ct2/show/NCT01452178. The hospital is the only referral center for a geographical area with around 180,000 inhabitants. 748 of 1015 eligible acute MI patients ≤80 years of age were individually sex- and age-matched with one control subject, without previous MI, selected from the population registry of the catchment area of the MI patients. The reasons for not matching an eligible patient are shown in a flow chart (Figure 1). All participants gave their informed consent. The final study cohort comprised 688 pairs with valid data for all anthropometric measures in both the case and the corresponding control. We performed age- and sex-stratified analyses. The age categories, 65 and below (≤65 yr) and 66 to 80 (≥66 yr), were chosen a priori. Sixty-five years is the usual age of retirement in Sweden.

### 2.2. Acute Myocardial Infarction Patients

ECG and troponin I criteria recommended by the European Society of Cardiology (ESC) were used for diagnosing acute MI [9]. Troponin I was analyzed with a radial partition immunoassay using the sandwich immunoassay principle (Stratus CS STAT, Dade Behring, Germany). The troponin I diagnostic limit of ≥400 ng/L, as recommended by ESC, was used at start of the study in the year 2005. During the recruitment period, this diagnostic limit was lowered to ≥100 ng/L due to changed recommendations. To obtain uniformity of the study population, we adhered to the original ≥400 ng/L limit. As troponin I levels reflect the degree of MI damage, the present study compares anthropometrics between patients with a relatively large MI and their control subjects. The MI diagnosis was founded on characteristic symptoms in combination with characteristic ECG pattern in 3% of patients with missing troponin I values.

### 2.3. Control Subjects

A preliminary study revealed major difficulties in recruiting controls >80 years of age. Thus, the study was restricted to individuals ≤80 years of age (69.5% of the 1459 patients in the VAMIS project), see flow chart, Figure 1. For every MI patient, one control subject of the same sex and with the nearest date of birth in the population registry of the catchment area of the cardiac center was selected, provided that this individual had not previously been diagnosed with MI. The study population was judged too small for matching on other variables than sex and age. The controls were recruited by telephone. For 61% of the MI patients, the first selected control agreed to participate. The time from arrival to the cardiac intensive care unit and inclusion in the study was 22 (16–40) hours (median (interquartile range)). The controls were examined in the research department at our hospital.

### 2.4. Measurements

Anthropometric measurements of patients were performed at inclusion of the patients in the study in the cardiac ward of the hospital. Only two nurses were involved in the anthropometric measurements of patients and controls. The two nurses controlled that their measurement techniques and results were consistent.

Body weight was measured to the nearest 0.1 kg with participants wearing underwear or light clothing. Height was measured to the nearest 1 cm. Waist was measured to the nearest 1 cm in the horizontal plane at the midpoint between the lowest rib and the iliac crest. Hip was measured to the nearest 1 cm around the widest portion of the buttocks. Each individual was measured once with a nonstretchable 1.5 m tape in standing position if the medical condition permitted. No case or control was excluded due to having too large circumference or being too heavy.

Self-reported diagnoses of previous MI, angina pectoris, stroke, diabetes, and drug-treated hypertension were verified from medical records. Current smoking was defined as daily smoking during the month before onset of acute MI or index examination of controls.

### 2.5. Statistics

Categorical variables were summarized as numbers and percentages (%). Continuous variables were summarized as means and standard deviations (SD) or, in the case of skewed distributions, by medians and interquartile ranges. Differences between cases and controls were assessed by the paired *t*-test for continuous and by McNemar's test for categorical variables. If the number of discordant pairs were 10 or fewer, an exact version of McNemar's test based on the binomial distribution was applied. Confidence intervals for unpaired categorical data were calculated according to Gardner and Altman [10].

Crude and adjusted associations between basic anthropometric measures and MI were assessed by conditional logistic regression for matched pairs. Nagelkerke's generalized *R* ^2^ was used to assess the variability explained by the model. A best subset approach with Schwarz Bayesian information criterion (BIC) was used to find the best conditional logistic regression models. BIC, which is a penalized version of the log likelihood function (the penalty is proportional to the number of parameters fitted), was chosen to avoid overfitting. The results were reported as odds ratios (OR) with 95% confidence intervals (CI).

The area under the receiver operating characteristic (AUROC) curve was used to assess the classification ability of individual and combined anthropometric measures.

Internal validation was performed using 10-fold cross-validation. The sample was split into 10 roughly equal-sized random parts, retaining the pair structure. Of the 10 subsamples, a single subsample was used as a validation set, and the remaining 9 subsamples were used to estimate the parameters of the model. This process was repeated 10 times, using each of the 10 subsamples once as validation set. Finally, the 10 resulting classification tables were merged. The best discriminating functions were derived by logistic regression analysis.

Feed forward neural network models with one hidden layer and a hyperbolic and a sigmoid activation function were assessed to check if the AUROC curves and the discriminating abilities could be markedly improved by including nonlinearities and interactions into the models.

A two-sided *P* value < 0.05 was regarded statistically significant. IBM SPSS Statistics 24 was used for all statistical analyses except the best subset conditional logistic regression analyses which were run in SAS 9.4.

## 3. Results

The study comprised 486 male and 202 female case-control pairs corresponding to 68% of eligible patients as illustrated in a flow chart (Figure 1). The mean (SD) age at inclusion of the patients was 65.3 (9.5) year, men 64.1 (9.5), and women 68.2 (8.9). The corresponding numbers for the control subjects were 66.1 (9.5) years, men 64.9 (9.5), and women 69.0 (8.9). The control subjects were 0.8 (0.3) years older than the MI patients at inclusion due to refusals to participate by some control subjects, necessitating selection of another control, or delays in examining the control subject for logistical reasons.

Table 1 and Supplementary Table 1 show clinical characteristics of cases and controls.  Table 2 shows the values of the anthropometric measures in cases and controls and the signed relative differences ((case-control)/control) for the different sex- and age-categories. The waist/hip was significantly larger in the patients; mainly due to a narrow hip in men and a large waist in women. Elderly female patients, as opposed to elderly male patients, had larger weight, height, BMI, waist, and hip than their controls. If restricted to only first time MI patients and their individually matched controls, the results remained essentially unchanged (Supplementary Table 2)

**Figure 2:**
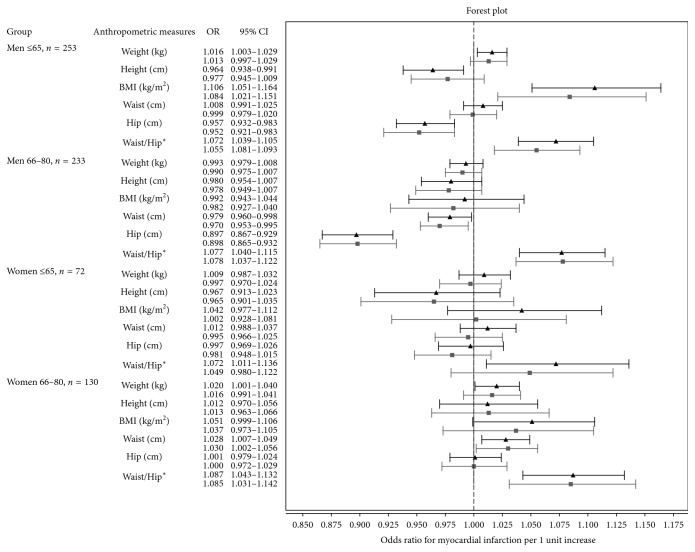
Forest plot illustrating the association between myocardial infarction status and basic anthropometric measures. Black whiskers with a triangle unadjusted and gray whiskers with a square adjusted for current smoking, anamnestic diabetes, drug-treated hypertension, angina pectoris, and stroke. ^*∗*^Waist/hip is per 1/100 unit.

Crude and adjusted associations of individual basic anthropometric measures and MI status were assessed by conditional logistic regression (Figure 2 and Supplementary Table 3). The strong reverse association between hip and MI status among men ≥66 yr stands out. For men ≤65 yr, this association was weaker but statistically significant. Waist/hip was the only anthropometric measure that was significantly associated with MI status in all sex- and age-categories.

The total variability in MI status explained by all anthropometric measures (weight, height, waist, hip, BMI, and waist/hip) simultaneously was 0.50 for men ≤65 yr, 0.62 for men ≥66 yr, 0.23 for women ≤65 yr, and 0.32 for women ≥66 yr as assessed by Nagelkerke's *R* ^2^.

**Figure 3:**
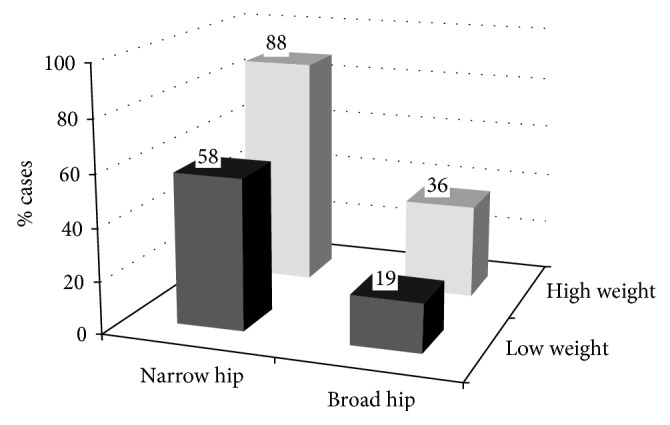
Distribution of myocardial infarction cases among men ≥66 yr categorized by below/above the medians of hip and weight for cases and controls taken together (103 cm and 80 kg) in men ≥66 yr (*n*=466). Numbers above the bars are percentage of cases. Narrow hip/Low weight (*n*=199; 116 cases, 83 controls), narrow hip/high weight (*n*=52; 46 cases, 6 controls), broad hip/low weight (*n*=37; 7 cases, 30 controls), and broad hip/high weight (*n*=178; 64 cases, 114 controls).

**Figure 4 fig4:**
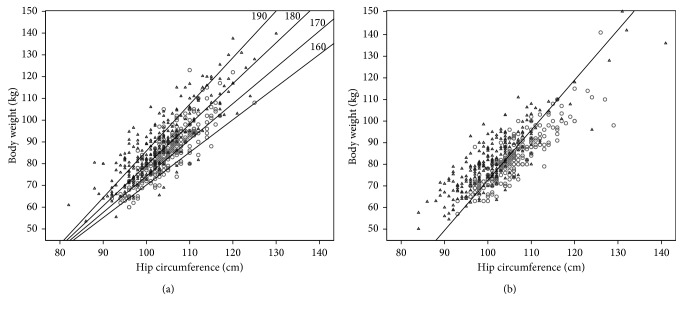
Scatter plot of weight versus hip in men ≤65 yr (a) and ≥66 yr (b). Triangles denote cases and circles denote controls. The straight line in (b) represents the optimal linear discrimination between cases and controls in men ≥66 yr, and the 4 nonparallel straight lines in (a) represent the optimal linear discrimination between cases and controls in men ≤65 yr for the heights: 160, 170, 180, and 190 cm.

**Figure 5:**
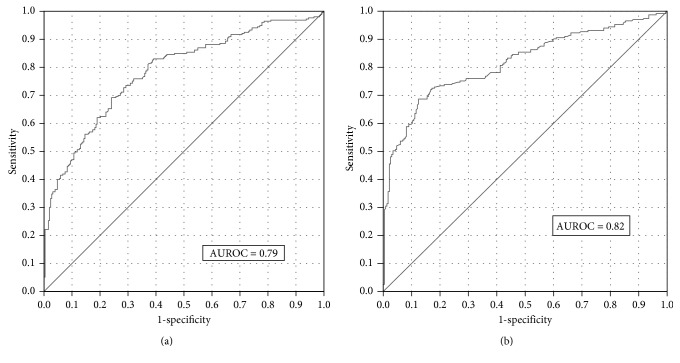
Receiver operating characteristic curves for best anthropometric subset models of myocardial infarction status in men ≤65 yr (a) (included variables: hip, BMI, and height) and men ≥66 yr (b) (included variables: hip and weight).

Best subset conditional logistic regression analyses were performed to find the combination of individual anthropometric measures which best predicted the MI status in the different sex- and age-categories (Supplementary Table 4). The ability of the combination of hip and weight to predict MI status among men ≥66 yr was especially strong (Figures 3 [Fig fig4]–5). The medians in cases and controls combined were 80 kg for weight and 103 cm for hip among men ≥66 yr. The proportion of MI cases among men ≥66 yr with a hip below (103 cm) and weight above 80 kg was 0.88 as compared to 0.45 in the three other hip weight combination groups taken together (Figure 3). The absolute difference between these proportions was 0.43 (95% CI 0.33–0.53). The category with a hip below the median and a weight above the median comprised 11% of all males ≥66 yr.  Figure 4 right panel illustrates the distribution of male MI patients ≥66 yr and their controls in a scatter plot of weight versus hip with the discriminating line (24.09 + 0.15 ∗ weight (kg) − 0.35 ∗ hip (cm)), which maximizes sensitivity + specificity based on logistic regression analyses. Using 10-fold cross-validation, we achieved a sensitivity of 0.72 and a specificity of 0.77.  Figure 4 left panel shows the corresponding scatter plot for men ≤65 yr with the discriminating plane (5.25 + 0.52 ∗ BMI (kg/m^2^) + 0.067 ∗ height (cm) − 0.30 ∗ hip (cm)) evaluated for the heights 160, 170, 180, and 190 cm, using the relation weight = BMI ∗ height^2^. Using 10-fold cross-validation, we achieved a sensitivity of 0.66 and a specificity of 0.72.

We also calculated AUROC curves for individual and combined anthropometric measures (Table 3, Figure 5, and Supplementary Figure 1). The rationale behind the selection of AUROC curves to present in Table 3 was as follows:

- All individual anthropometric measures were to be presented.
- The combination of waist and hip was included since it was much more informative than waist/hip in males according to the BIC.
- The combinations of BMI, hip, and height; weight and hip; and BMI and hip were the best in men ≤65 yr, men ≥66 yr, and women ≤65 yr, respectively, according to the BIC.
- The combination of weight, hip, and height was included since it was the best combination taken over all four sex/age categories according to the BIC.

The largest AUROC curve for an individual anthropometric measure in any category was found for hip among men ≥66 yr. The AUROC curve for the combination of hip and weight in men ≥66 yr was remarkably large (0.82). Notably, the AUROC curve for a model including hip and waist was larger than for a model including waist/hip alone except for women ≥66 yr (Table 3). If restricted to only first time MI patients and their individually matched controls, the AUROC curves remained essentially unchanged (Supplementary Table 5).

In addition to the logistic regression models used to estimate the AUROC curves, we also assessed feed forward neural network models with one hidden layer and a hyperbolic tangent and a sigmoid activation function. These models can model both nonlinearities and interactions. Applying the neural network models only marginally increased the AUROC curves: men ≥66 yr (weight, hip) from 0.82 to 0.83 and men ≤65 yr (BMI, hip, and height) from 0.79 to 0.80. Since the gain was very marginal, it was decided to only present the results from the logistic regression analyses.

## 4. Discussion

The most striking finding in the present study was the high proportion of MI among elderly men with a narrow hip and a disproportionally high weight (0.88). We are not aware of any previous data on this topic. Our observation that a large waist/hip was related to MI in all sex- and age-categories, confirms present knowledge.

There are few case-control studies concerning acute MI [5–8]. The study by Yusuf et al. [5] is by far the largest one comprising 27,000 participants from 52 countries. The controls were sex- and age-matched and without a history of cardiovascular disease. They comprised mainly attendants or relatives of patients from a noncardiac ward or an unrelated attendant of another cardiac patient. A trend towards higher risk for MI as hip circumference decreased was found, which is consistent with our findings. They also found waist/hip to be a stronger risk factor for MI than waist or BMI. Similar results have been reported from case-control studies by Kahn et al. [6], Azevedo et al. [7], and Oliveira et al. [8] using groups of randomly selected controls from the catchment areas of the patients.

In contrast to case-control studies of acute MI, there are many prospective studies concerning the relations between anthropometric measures in general populations and future outcomes such as all-cause mortality [4, 11–20] and cardiovascular events, including MI [2, 3, 13, 16, 21, 22]. A consistent finding in these studies was that an excess of body fat as reflected by large BMI and particularly by large waist and waist/hip predicted future adverse cardiac events. Notably, the combination of large waist and narrow hip is especially useful to predict future MI according to an extensive review of prospective studies from general populations by Cameron et al. [13].

### 4.1. Narrow Hip as a Risk Factor in Prospective Follow-Up Studies

Heitmann and Lissner [23] presented a summary of 13 reports about prospective follow-up studies of hip as predictors of heart disease and total mortality. Most reports showed an association between a narrow hip and adverse outcomes in both sexes. Further, Heitmann et al. [15] reported from a prospective observational study, with anthropometric measures determined between 35 and 65 years of age, that a large hip relative to BMI and waist predicted less incidence of cardiovascular disease, coronary heart disease, and all-cause mortality in women but not in men. Lissner et al. [24] reported that the 24-year incidence of MI as well as all-cause, cardiovascular, and MI mortality was inversely associated with hip in women. Cameron et al. [12] reported from a large population-based survey that waist circumference was strongly related to mortality after adjustment for hip and vice versa.

The hip is affected by the dimension of the pelvic girdle, subcutaneous fat, and the gluteal muscles. The gluteal muscles constitute the largest muscle group in the body and are reasonably a major determinant of hip circumference. Therefore, the relation between MI and a narrow hip among elderly men in the present study suggests an association between MI and age-related loss of muscle mass, that is, sarcopenia [25]. Reasonably, hip circumference reflects muscle mass better than the other anthropometric measures in the present study.

### 4.2. Does Waist/Hip Fully Reflect Waist and Hip as Risk Factors?

In men, the AUROC curve of hip and waist in the same model is larger than in a model including waist/hip alone. Thus, the full strength of the association between waist, hip, and MI risk was not fully reflected by waist/hip but became apparent when both waist and hip were included in the same model. This finding is consistent with a systematic literature review of prospective studies of anthropometric measures [13].

### 4.3. Principal Difference between Prospective Cohort and Case-Control Studies

In prospective cohort studies, the risk factors in future event subjects and nonevent subjects are typically registered several years before the event. Contrarily, the risk factors in matched case-control studies are, in both cases and controls, registered at about the time as the index event. This implies a fundamental difference from prospective follow-up studies that register phenotypes of future cases and noncases at a considerable time, usually several years, before the event. The difference in the life trajectory of an anthropometric measure, such as weight, between case and control reasonably varies over the life span. There is an abundance of data on the relationship between anthropometric measures from several years before the patient had a MI but very few anthropometric data from the period close before. Anthropometric measures may change significantly during these years. Consequently, the relations between the levels of potential risk factors in cases and controls may differ considerably between case-control and prospective studies. For example, a difference in hip registered in an MI case-control study may depend on decreasing hip in cases but not in their controls during the years before the MI event. Thus, studies of development of anthropometric measures over time in the same individual are of great importance.

Hughes et al. [26] studied 10-year development of the same anthropometric measures as in our study among 35 men with a baseline mean age of 60. They found a 10-year mean decrease in hip from 100.5 cm to 97.8 cm. In terms of both absolute and percentage, this was clearly larger than the increase in waist from 91.4 to 92.1 cm. Mousavi et al. [27] studied the associations between mortality and changes of BMI, waist, hip, and waist/hip over 7 years in 1805 Iranian men free from cardiovascular diseases at baseline. A positive association between mortality and decrease in hip as well as between mortality and increase in waist/hip was the only statistically significant association. Analogously, it is likely that decreasing hip in future MI patients but not in the control subjects over the years before the MI event is related to the considerable case-control difference in our study.

Since the anthropometric measures in acute MI patients are reasonably valid at least for a few months before the MI event, the case-control study may be more suitable than a prospective cohort study to examine the relations between MI status and anthropometric measures.

### 4.4. Strengths and Limitations

Strength of the present study was the clearly defined recruitment of the cases and controls, which enabled us to select the controls in an unbiased fashion. This strength is, however, partly offset by the difficulty in generalizing our findings to those from other geographical areas. A further possible limitation is the lack of consensus regarding the optimal protocol for measuring hip circumference. Regarding waist, a systematic review has shown that variation of waist measurement protocols has no substantial influence on the associations between waist and different outcomes found in prospective studies, but there are no corresponding analyses concerning hip [28].

An inevitable limitation of the case-control design in the present setting is that data from patients dying before hospitalization are unobtainable, which might cause a minor selection bias.

### 4.5. Clinical Implications

Our finding that a phenotype with a narrow hip combined with a disproportionally high weight is associated with MI among elderly men may be clinically useful, but further studies in similar populations are needed. Non-MI patients with this phenotype may deserve attention for undiagnosed cardiac disease. Recommendations for appropriate nutrition and physical activity to maintain muscle mass as well as research on the relations between dietary habits and anthropometric phenotypes are required.

## 5. Conclusions

The most noteworthy finding in this study was the evidently narrower hip in the elderly male MI patients compared to their controls. This raises the suspicion of smaller gluteal muscles, due to more advanced sarcopenia, in elderly male MI patients. Such sarcopenia may reflect general aging.

In general, over all four sex- and age-categories, a phenotype characterized by the combination narrow hip circumference, high body weight, and short length was the most highly associated with an increased risk of myocardial infarction (area under the receiver operating curve: men ≤65 yr = 0.79, men ≥66 yr = 0.82, women ≤65 yr = 0.68, and women ≥66 yr = 0.67). This also points on the striking finding that the association between anthropometric measures and myocardial infarction is much stronger in males than in females.

## Figures and Tables

**Table 1:** Pertinent clinical characteristics categorized by case-control status within each sex and age (≤65/≥66 years) stratum.

	Men ≤65 yr (*n*=253)	Men ≥66 yr (*n*=233)	Women ≤65 yr (*n*=72)	Women ≥66 yr (*n*=130)
Anamnestic diabetes	41 (16)/16 (6)	41 (18)/27 (12)	11 (15)/4 (6)	24 (18)/14 (11)
	*P*=0.001	*P*=0.081	*P*=0.118	*P*=0.110

Drug-treated hypertension	111 (44)/60 (24)	113 (49)/110 (47)	24 (33)/13 (18)	79 (61)/69 (53)
	*P* < 0.001	*P* > 0.20	*P*=0.043	*P* > 0.20

Current smoker	85 (34)/34 (13)	27 (12)/17 (7)	28 (39)/14 (19)	24 (19)/6 (5)
	*P* < 0.001	*P*=0.143	*P*=0.020	*P*=0.001

Angina pectoris	31 (12)/4 (2)	73 (31)/26 (11)	8 (11)/0 (0)	42 (32)/9 (7)
	*P* < 0.001	*P* < 0.001	*P*=0.008	*P* < 0.001

Previous stroke	6 (2)/7 (3)	20 (9)/26 (11)	0 (0)/2 (3)	14 (11)/14 (11)
	*P* > 0.20	*P* > 0.20	*P* > 0.20	*P* > 0.20

First time MI	212 (84)/0^a^	172 (74)/0^a^	64 (89)/0^a^	100 (77)/0^a^

Obesity (BMI ≥30.0)	69 (27)/39 (15)	37 (16)/41 (18)	22 (31)/14 (19)	35 (27)/24 (18)
	*P*=0.001	*P* > 0.20	*P*=0.185	*P*=0.135

^a^Controls have per definition no prior MI. Figures are number (%) for cases/controls.

**Table 2:** Anthropometric measures: means (SD) and signed relative difference ((case-control)/control) in per cent between myocardial infarction cases and their matched controls.

	Weight (kg)	Height (cm)	BMI	Waist (cm)	Hip (cm)	Waist/hip
Men ≤65 yr; *n*=253						
Cases	88.9 (15.6)	177.3 (6.9)	28.2 (4.4)	100.8 (11.5)	103.1 (8.3)	0.98 (0.07)
Controls	85.9 (11.9)	178.8 (6.5)	26.8 (3.2)	99.9 (9.6)	105.1 (5.5)	0.95 (0.06)
*P*	0.017	0.008	<0.001	>0.20	0.001	<0.001
Signed relative difference	+3.5	−0.8	+5.2	+1.0	−1.9	+3.2

Men ≥66 yr; *n*=233						
Cases	81.6 (14.4)	175.2 (6.9)	26.5 (4.0)	98.8 (10.4)	100.9 (7.7)	0.98 (0.06)
Controls	82.5 (11.8)	176.1 (6.9)	26.6 (3.5)	100.8 (9.8)	105.6 (6.2)	0.95 (0.06)
*P*	>0.20	0.138	>0.20	0.023	<0.001	<0.001
Signed relative difference	−1.1	−0.6	−0.5	−2.0	−4.5	+3.2

Women ≤65 yr; *n*=72						
Cases	73.4 (16.7)	162.9 (5.9)	27.6 (6.0)	92.7 (14.6)	105.3 (12.6)	0.88 (0.07)
Controls	71.5 (12.7)	164.1 (5.7)	26.5 (4.4)	90.4 (13.3)	105.6 (9.4)	0.85 (0.07)
*P*	>0.20	>0.20	>0.20	>0.20	>0.20	0.014
Signed relative difference	+2.7	−0.7	+4.2	+2.5	−0.3	+3.5

Women ≥66 yr; *n*=130						
Cases	73.3 (15.7)	162.2 (6.2)	27.9 (6.4)	95.0 (13.6)	105.6 (12.2)	0.90 (0.07)
Controls	69.8 (13.2)	161.8 (6.0)	26.6 (4.6)	90.6 (12.1)	105.5 (10.6)	0.86 (0.07)
*P*	0.038	>0.20	0.048	0.006	>0.20	<0.001
Signed relative difference	+5.0	+0.2	+4.9	+4.9	0.0	+4.7

*P* value refers to the case-control difference.

**Table 3:** Individual and combined anthropometric measures' ability to discriminate MI cases and control subjects expressed as AUROC curve.

Measure	AUROC (95% CI)	Direction of association^a^	*P*
Men ≤65 yr; *n*=253			
Weight	0.54 (0.49–0.59)	+	0.091
Height	0.56 (0.51–0.61)	−	0.010
Waist	0.50 (0.45–0.55)	+	>0.20
Hip	**0.60** (0.56–0.65)	−	<0.001
BMI	0.59 (0.54–0.64)	+	0.001
Waist/hip	**0.62** (0.57–0.67)	+	<0.001
Waist and hip	**0.67** (0.62–0.72)	+, −	<0.001
Weight and hip	**0.76** (0.72–0.80)	+, −	<0.001
BMI and hip	**0.77** (0.73–0.81)	+, −	<0.001
Weight, hip, and height^c^	**0.79** (0.75–0.83)	+, −, −	<0.001
BMI, hip, and height^b,d^	**0.79** (0.75–0.83)	+, −, +	<0.001

Men ≥66 yr; *n*=233			
Weight	0.53 (0.48–0.59)	−	>0.20
Height	0.54 (0.49–0.59)	−	0.139
Waist	0.56 (0.51–0.61)	−	0.021
Hip	**0.72** (0.67–0.76)	−	<0.001
BMI	0.52 (0.47–0.57)	−	>0.20
Waist/hip	**0.61** (0.57–0.67)	+	<0.001
Waist and hip	**0.77** (0.72–0.81)	+, −	<0.001
Weight and hip^b^	**0.82** (0.78–0.86)	+, −	<0.001
BMI and hip	**0.78** (0.74–0.83)	+, −	<0.001
Weight, hip, and height^c^	**0.82** (0.78–0.86)	+, −, −	<0.001
BMI, hip, and height^d^	**0.81** (0.77–0.85)	+, −, +	<0.001

Women ≤65 yr; *n*=72			
Weight	0.52 (0.42–0.61)	+	>0.20
Height	0.55 (0.45–0.64)	−	>0.20
Waist	0.55 (0.45–0.64)	+	>0.20
Hip	0.54 (0.44–0.63)	−	>0.20
BMI	0.55 (0.45–0.64)	+	>0.20
Waist/hip	**0.61** (0.52–0.71)	+	0.019
Waist and hip	**0.62** (0.53–0.71)	+, −	0.011
Weight and hip	**0.64** (0.54–0.73)	+, −	0.005
BMI and hip^b^	**0.68** (0.58–0.76)	+, −	<0.001
Weight, hip, and height^c^	**0.68** (0.59–0.77)	+, −, −	<0.001
BMI, hip, and height^d^	**0.68** (0.60–0.77)	+, −, +	<0.001

Women ≥66 yr; *n*=130			
Body weight	0.57 (0.50–0.64)	+	0.048
Height	0.52 (0.45–0.59)	+	>0.20
Waist	0.59 (0.52–0.66)	+	0.009
Hip	0.53 (0.46–0.60)	+	>0.20
BMI	0.56 (0.49–0.63)	+	0.120
Waist/hip^b^	**0.67** (0.61–0.74)	+	<0.001
Waist and hip	**0.67** (0.61–0.74)	+, −	<0.001
Weight and hip	**0.66** (0.59–0.73)	+, −	<0.001
BMI and hip	**0.64** (0.58–0.71)	+, −	<0.001
Weight, hip, and height^c^	**0.67** (0.60–0.74)	+, −, −	<0.001
BMI, hip, and height^d^	**0.67** (0.60–0.74)	+, −, +	<0.001

AUROC curve values larger than or equal 0.60 are in bold. ^a^“+” denotes that a larger value of the anthropometric measure increases the risk of MI, and “−” denotes that a large value decreases the risk. ^b^Best subset of anthropometric measures according to Schwarz Bayesian information criterion in the individual sex/age categories. ^c^Best subset of anthropometric measures according to Schwarz Bayesian information criterion over all four individual sex/age categories. ^d^The “+” direction of the height effect is only apparent. The true direction is negative since it appears in the denominator of BMI.

## Data Availability

The data used to support the findings of this study are available from the corresponding author upon request.

## Supplementary Materials

Supplementary Materials The supplementary material section contains 5 tables and 1 figure. Supplementary Table 1 is a more comprehensive version of Table 1. Supplementary Table 2 corresponds to Table 2 but includes only first time MI cases and their matched controls. Supplementary Table 3 contains the numbers for Figure 1. Supplementary Table 4 contains the result of the best subset conditional logistic regression analyses for finding the combination of individual anthropometric measures which best predicted the MI status in the different sex- and age-categories. Supplementary Table 5 corresponds to Table 3 but includes only first time MI cases and their matched controls. Supplementary Figure 1 corresponds to Figure 5 and shows the area under the operating characteristic curves for the best subset of anthropometric measures for women.
