# Corrigendum to “Basic Anthropometric Measures in Acute Myocardial Infarction Patients and Individually Sex- and Age-Matched Controls from the General Population”

**DOI:** 10.1155/2018/3126805

**Published:** 2018-12-18

**Authors:** Göran Nilsson, Pär Hedberg, Jerzy Leppert, John Ohrvik

**Affiliations:** ^1^Center for Clinical Research, Region Vastmanland-Uppsala University, Hospital of Vastmanland, Västerås, Sweden; ^2^Department of Clinical Physiology, Hospital of Vastmanland, Västerås, Sweden

In the article titled “Basic Anthropometric Measures in Acute Myocardial Infarction Patients and Individually Sex- and Age-Matched Controls from the General Population” [1], Dr. Andreas Rosenblad was incorrectly included as an author. The corrected authors' list is shown above and updated in place. In addition, the Acknowledgements section is updated in the article to be as follows.

The authors are grateful to Marja-Leena Ojutkangas and Annika Kärnsund for their excellent care of the study participants and their careful data management. The authors are also grateful to Dr. Andreas Rosenblad for taking part in the design of the VAMIS study and critical review regarding the intellectual content of earlier versions of the paper and Mattias Rehn for skillful technical support and data management. This study received grants from Sparbanksstiftelsen Nya and the Västmanland County Council Research Foundation.
