# Supplementary material for: Basic Anthropometric Measures in Acute Myocardial Infarction Patients and Individually Sex- and Age-Matched Controls from the General Population
Source: J Obes. 2018 Oct 2;2018:3839482. doi: 10.1155/2018/3839482 (PMC6247440; doi:10.1155/2018/3839482)

Supplementary Material

Supplementary Table 1. Basic characteristics of the VAMIS participants by sex.

	Men (n=486)		Women (n=202)		
	Cases	Controls	Cases	Controls	Missing values %
**Anthropometric Variables**					
Weight, kg	85.4(15.4)	84.3(12.0)	73.3(16.0)	70.4(13.0)	0/0/0/0
Height, cm	176.3(7.0)	177.5(6.8)	162.5(6.1)	162.6(6.0)	0/0/0/0
BMI, kg/m^2	27.4(4.3)	26.7(3,4)	27.9(6.2)	26.6(4.5)	0/0/0/0
Waist, cm	99.8(11.0)	100.3(9.7)	94.2(14.0)	90.5(12.5)	0/0/0/0
Hip, cm	102.0(8.1)	105.4(5.9)	105.5(12.3)	105.5(10.2)	0/0/0/0
Waist/hip	0.98(0.07)	0.95(0.06)	0.89(0.07)	0.86(0.07)	0/0/0/0
**Biochemical Variables**					
Cholesterol, mmol/L[a]	5.0(1.3)	5.5(1.1)	5,2(1.4)	6.0(1.1)	0/1/0/1
LDL-cholesterol, mmol/L[a]	3.2(1.2)	3.6(1.0)	3.2(1.1)	3.8(1.0)	5/3/5/3
HDL-cholesterol, mmol/L[a]	1.04(0.31)	1.23(0.35)	1.28(0.41)	1.61(0,46)	0/1/0/1
Triglycerides, mmol/L[b]	1.8(1.2)	1.4(0.96)	1.6(1.1)	1.3(0.9)	0/1/0/1
Fasting plasma glucose, mmol/L[c]	5.9(5.3-6.8)	5.9(5.4-6.5)	6.0(5.4-6.8)	5.6(5.2-6.3)	3/1/2/2
Max Troponin I, ng/L	8560(1960-30080)	NA	4910(1655-22560)	NA	3/-/3/-
CRP top quartile (>6.0 mg/mL)	151(32)	37(8)	82(43)	20(10)	4/1/5/2
Leukocytes, 10^9/L	10.0(3.5)	6.6(1.8)	10.6(4.0)	6.3(1.7)	0/1/1/1
**History of**					
Diabetes Mellitus	82(17)	43(9)	35(17)	18(9)	0/0/0/0
Hypercholesterolemia	161(33)	139(29)	68(34)	50(25)	0/0/0/0
Hypertension	267(55)	214(44)	118(58)	101(50)	0/0/0/0
Angina pectoris	104(21)	30(6)	50(25)	9(4)	0/0/0/0
Myocardial Infarction	102(21)	NA	38(19)	NA	0/-/0/-
Heart failure	28(6)	11(2)	15(7)	2(1)	0/0/0/0
Prior stroke	26(5)	33(7)	14(7)	16(8)	0/0/0/0
**Medication**					
Glucose lowering drugs (insulin/analogs or peroral drugs)	71(15)	33(7)	32(16)	12(6)	0/0/0/0
Insulin/analogs	33((7)	13(3)	19(9)	5(2)	0/0/0/0
Statins	148(30)	90(18)	62(31)	36(18)	0/0/0/0

Non-statins lipid-lowering drugs	1(0)	3(1)	0	0	0/0/0/0
Anti-hypertensive drugs	224(46)	170(35)	103(51)	82(41)	0/0/0/0
Acetylicsalicylic acid	156(32)	89(18)	70(35)	36(18)	0/0/0/0
Anticoagulants	19(4)	26(5)	5(2)	4(2)	1/0/1/0
Anti-platelets drugs	15(3)	6(1)	13(6)	0	0/0/0/0
**Miscellaneous variables**					
Hours from symptom onset to inclusion	32(24-51)	32(24-53)	30(22-43)	36(25-62)	19/22/24/18
ST-elevation myocardial infarction	138(28)	NA	49(24)	NA	0/-/0/-
Obesity (BMI ≥ 30)	106(22)	80(16)	57(28)	38(19)	0/0/0/0
Obesity/overweight (BMI ≥ 25)	333(69)	132(27)	329(68)	112(55)	0/0/0/0
Current smokers	112(23)	51(10)	52(26)	20(10)	0/0/0/0
Never smokers	139(29)	198(41)	76(38)	103(51)	0/0/0/0
Current tobacco snuffers	56(12)	69(7)	3(2)	3(2)	3/0/4/1
Never tobacco snuffers	357(76)	351(72)	3(2)	3(2)	3/0/4/1
Married/cohabitant	376(77)	408(84)	127(63)	128(63)	0/0/0/0

Values are expressed as mean (SD), median (interquartile range) or number (%). Abbreviations: BMI = body mass index; HDL-cholesterol = high-density lipoprotein cholesterol; LDL-cholesterol = low-density lipoprotein cholesterol; CRP = C-reactive protein; NA = not applicable.

[a] To convert the values for cholesterol from mmol/L to mg/dL multiply by 38.67.

[b] To convert the values for triglycerides from mmol/L to mg/dL multiply by 88.57.

[c] To convert the values for glucose from mmol/L to mg/dL multiply by 18.02.

Supplementary Table 2. Anthropometric measures: means (SD) and signed relative difference ([case – control]/control) in per cent between first time myocardial infarction cases and their matched controls.

	Weight	Height	BMI	Waist	Hip	Waist/hip
Men ≤65yrs; n = 212						
Cases	89.0(14.9)	177.5(7.0)	28.2(4.1)	100.5(11.0)	103.1(7.5)	0.97(0.06)
Controls	86.4(11.9)	178.8(6.5)	27.0(3.2)	100.1(9.6)	105.3(5.5)	0.95(0.06)
P	0.047	0.046	0.001	>0.20	0.001	< 0.001
Signed relative difference	+3.0	-1.0	+4.4	0.0	-2.1	+2.1
Men ≥66yrs; n = 172						
Cases	81.8(14.9)	175.4(6.9)	26.5(4.0)	98.7(10.7)	101.0(7.9)	0.98(0.06)
Controls	82.7(11.6)	176.2(7.0)	26.7(3.6)	100.8(9.9)	105.6(6.2)	0.95(0.06)
P	>0.20	>0.20	>0.20	0.062	< 0.001	< 0.001
Signed relative difference	-1.1	-0.5	-0.7	-2.1	-4.4	+3.2
Women ≤65yrs; n = 64						
Cases	72.0(15.5)	162.8(5.9)	27.1(5.4)	91.2(13.6)	104.1(11.6)	0.87(0.07)
Controls	71.8(13.3)	163.8(5.8)	26.7(4.6)	90.9(13.8)	106.0(9.9)	0.85(0.07)
P	>0.20	>0.20	>0.20	>0.20	>0.20	0.073
Signed relative difference	0.0	-0.1	+0.1	+0.2	-2.8	+2.4
Women ≥66yrs; n = 100						
Cases	72.1(14.9)	162.4(6.1)	27.3(5.6)	93.9(13.7)	105.0(11.3)	0.89(0.07)
Controls	70.1(13.2)	162.7(5.8)	26.4(4.6)	90.2(12.1)	105.5(11.1)	0.86(0.07)
P	>0.20	>0.20	>0.20	0.048	>0.20	< 0.001
Signed relative difference	+2.9	-0.1	+3.4	+4.1	0.0	+3.5

P refers to case-control difference.

Supplementary Table 3. Crude and adjusted association between myocardial infarction status and basic anthropometric measures assessed by conditional logistic regression

	Crude model			Adjusted model[a]		
	ln(OR)	OR(95%CI)	*P*-value	ln(OR)	OR(95%CI)	*P*-value
Men ≤65yrs; n = 253						
Weight, kg	0.02	1.02(1.00–1.03)	0.016	0.01	1.01(1.00–1.03)	0.11
Height, cm	-0.04	0.97(0.94–0.99)	0.010	-0.02	0.98(0.95–1.01)	0.11
BMI, kg/m^2	0.10	1.11(1.05–1.17)	< 0.001	0.08	1.09(1.02–1.15)	0.007
Waist, cm	0.01	1.01(0.99–1.04)	>0.20	0.00	1.00(0.98–1.02)	>0.20
Hip, cm	-0.05	0.96(0.93–0.98)	0.002	-0.05	0.95(0.92–0.98)	0.003
Waist/hip[b]	0.07	1.07(1.03–1.11)	< 0.001	0.05	1.05(1.02–1.09)	0.005
Men ≥66yrs; n = 233						
Weight, kg	-0.01	0.99(0.98–1.01)	>0.20	-0.01	0.99(0.98–1.01)	>0.20
Height, cm	-0.02	0.98(0.96–1.01)	0.17	-0.02	0.98(0.95–1.01)	0.14
BMI, kg/m^2	-0.01	0.99(0.94–1.04)	>0.20	-0.02	0.98(0.93–1.04)	>0.20
Waist, cm	-0.02	0.98(0.96–1.00)	0.028	-0.03	0.97(0.95–1.00)	0.018
Hip, cm	-0.11	0.90(0.87–0.93)	< 0.001	-0.11	0.90(0.87–0.93)	< 0.001
Waist/hip[b]	0.07	1.08(1.04–1.11)	< 0.001	0.08	1.08(1.04–1.12)	< 0.001
Women ≤65yrs; n =72						
Weight, kg	0.00	1.01(0.99–1.03)	>0.20	0.00	1.00(0.97–1.03)	>0.20
Height, cm	-0.03	0.97(0.91–1.02)	>0.20	-0.04	0.97(0.90–1.04)	>0.20
BMI, kg/m^2	0.05	1.05(0.98–1.12)	0.16	0.01	1.01(0.93–1.09)	>0.20
Waist, cm	0.01	1.01(0.99–1.04)	>0.20	-0.01	1.00(0.97–1.03)	>0.20
Hip, cm	0.00	1.00(0.97–1.03)	>0.20	-0.02	0.98(0.95–1.02)	>0.20
Waist/hip[b]	0.07	1.07(1.01–1.14)	0.019	0.05	1.05(0.98–1.12)	0.17
Women ≥66yrs; n=130						
Weight, kg	0.02	1.02(1.00–1.04)	0.043	0.02	1.02(0.99–1.04)	>0.20
Height, cm	0.01	1.01(0.97–1.06)	>0.20	0.01	1.01(0.96–1.07)	>0.20
BMI, kg/m^2	0.05	1.05(1.00–1.11)	0.055	0.04	1.04(0.97–1.11)	>0.20
Waist, cm	0.03	1.03(1.01–1.05)	0.008	0.03	1.03(1.00–1.06)	0.018
Hip, cm	0.00	1.00(0.98–1.02)	>0.20	0.00	1.00(0.97–1.03)	>0.20
Waist/hip[b]	0.08	1.09(1.04–1.13)	<0.001	0.08	1.09(1.03–1.14)	0.002

[a] Adjusted for current smoking, diabetes, drug-treated hypertension, angina pectoris and stroke.

[b] Waist/hip per 1/100 unit.

Supplementary Table 4. Best conditional logistic regression models of the association between myocardial infarction status and basic anthropometric measures for each sex and age category based on the Bayesian information criterion (BIC). Within each category the 7 best models have been ordered with respect to the BIC.

	BIC	Measure	ln(OR)	Wald X^2	OR (95% CI)	*P*-value
Men ≤65yrs; n =253	251.6	Height cm	0.06	8.1	1.07 (1.02–1.11)	0.004
		BMI kg/m^2	0.50	59.1	1.66 (1.46–1.88)	< 0.001
		Hip cm	-0.29	53.9	0.75 (0.69–0.81)	< 0.001
	253.6	Height cm	-0.09	17.5	0.92(0.88–0.95)	<0.001
		Weight kg	0.15	58.8	1.17(1.12–1.21)	<0.001
		Hip cm	-0.28	53.7	0.76(0.70–0.82)	<0.001
	254.6	BMI kg/m^2	0.43	58.1	1.54(1.38–1.72)	0.001
		Hip cm	-0.24	53.6	0.79(0.74–0.84)	<0.001
	268.8	Weight kg	0.13	51.6	1.14(1.10–1.18)	<0.001
		Hip cm	-0.26	53.7	0.77(0.72–0.82)	<0.001
	319.3	Waist cm	0.09	26.2	1.09(1.05–1.13)	<0.001
		Hip cm	-0.15	32.5	0.86(0.82–0.91)	<0.001
	323.6	Height cm	-0.02	1.24	0.98(0.95–1.01)	>0.20
		Waist cm	0.08	25.6	1.08(1.05–1.12)	<0.001
		Hip cm	-0.13	27.3	0.87(0.83–0.92)	<0.001
	333.4	Waist/hip[a]	0.07	19.5	1.07(1.03–1.11)	<0.001
Men ≥66yrs; n = 233	204.0	Weight kg	0.16	48.7	1.17 (1.12–1.23)	< 0.001
		Hip cm	-0.36	62.9	0.70 (0.64–0.77)	< 0.001
	208.1	Height cm	-0.02	1.33	0.98 (0.94–1.02)	>0.20
		Weight kg	0.17	48.7	1.18 (1.13–1.24)	< 0.001
		Hip cm	-0.36	63.0	0.70 (0.64–0.77)	< 0.001
	216.5	Height cm	0.12	8.13	1.13(1.08–1.19)	<0.001

		BMI kg/m[2]	0.49	45.3	1.63(1.42–1.87)	<0.001
		Hip cm	-0.33	61.5	0.72(0.66–0.78)	<0.001
	240.4	BMI kg/m[2]	0.29	32.5	1.34(1.21–1.49)	<0.001
		Hip cm	-0.22	55.7	0.80(0.76–0.85)	<0.001
	255.7	Waist cm	0.10	21.6	1.10(1.06–1.15)	<0.001
		Hip cm	-0.21	47.2	0.81(0.76–0.86)	<0.001
	258.3	Height cm	0.03	2.9	1.03(1.00–1.07)	0.091
		Waist cm	0.10	22.9	1.11(1.06–1.15)	<0.001
		Hip cm	-0.23	48.5	0.80(0.75–0.85)	<0.001
	308.1	Waist/hip[a]	0.07	17.5	1.07(1.03–1.11)	<0.001
Women ≤65yrs; n = 72	92.7	BMI kg/m[2]	0.35	11.8	1.42(1.16–1.73)	<0.001
		Hip cm	-0.15	10.7	0.86(0.79–0.94)	0.001
	96.7	Height cm	0.01	0.05	1.01 (0.94–1.08)	>0.20
		BMI kg/m[2]	0.28	8.0	1.32 (1.09–1.59)	< 0.005
		Hip cm	-0.12	6.9	0.89 (0.82–0.97)	< 0.008
	98.0	Waist/hip[a]	0.07	5.5	1.07 (1.01–1.14)	0.019
	101.4	Height cm	-0.09	5.5	0.92(0.85–0.99)	0.018
		Weight kg	0.10	8.0	1.11(1.03–1.16)	0.005
		Hip cm	-0.12	7.0	0.89(0.82–0.96)	0.008
	101.7	Waist cm	0.07	5.9	1.08(1.01–1.14)	0.015
		Hip cm	-0.08	5.2	0.92(0.86–0.99)	0.023
	103.4	Weight kg	0.06	5.9	1.06 (1.00–1.13)	0.035
		Hip cm	-0.07	3.9	0.93 (0.87–1.00)	0.048
	105.0	Height cm	-0.03	0.98	0.97(0.92–1.03)	>0.20
		Waist cm	0.07	4.7	1.07(1.01–1.14)	0.018
		Hip cm	-0.08	5.6	0.93(0.86–0.96)	0.031

Women ≥66yrs; n = 130	165.1	Waist/hip[a]	0.08	16.0	1.09 (1.04–1.13)	< 0.001
	167.6	Waist cm	0.09	17.8	1.09(1.05–1.14)	< 0.001
		Hip cm	-0.09	12.3	0.92(0.88–0.96)	< 0.001
	172.2	Height cm	0.01	0.23	1.01(0.97–1.06)	>0.20
		Waist cm	0.09	17.7	1.09(1.05–1.14)	< 0.001
		Hip cm	-0.09	12.3	0.92(0.88–0.96)	< 0.001
	172.9	Weight kg	0.09	13.7	1.09 (1.04–1.14)	< 0.001
		Hip cm	-0.09	10.5	0.91 (0.86–0.96)	0.001
	175.2	BMI kg/m^2	0.20	11.6	1.23(1.09–1.38)	<0.001
		Hip cm	-0.09	8.8	0.92(0.87–0.97)	0.003
	175.6	Height cm	0.06	5.4	1.01 (0.94–1.08)	>0.20
		BMI kg/m^2	0.26	15.6	1.32 (1.09–1.59)	0.005
		Hip cm	-0.11	12.4	0.89 (0.82–0.97)	0.008
	176.6	Height cm	-0.03	1.2	0.97(0.92–1.02)	>0.20
		Weight kg	0.10	14.0	1.10(1.05–1.16)	0.001
		Hip cm	-0.11	11.1	0.90(0.85–0.96)	<0.001

[a] Waist/hip per 1/100 unit

Supplementary Table 5. Individual and combined anthropometric measures' ability to discriminate first time myocardial infarction cases and their matched controls expressed as area under the ROC curve.

Measure	AUROC (95%CI)	Direction of association[a]	*P*
Men ≤65yrs; n=212			
Weight	0.54(0.48–0.59)	+	0.18
Height	0.57(0.52–0.63)	–	0.011
Waist	0.49(0.44–0.55)	+	>0.20
Hip	**0.61**(0.56–0.67)	–	<0.001
BMI	0.59(0.53–0.64)	+	0.002
Waist/hip	**0.61**(0.56–0.68)	+	<0.001
Waist and hip	**0.67**(0.62–0.73)	+ , –	<0.001
Weight and hip	**0.76**(0.71–0.80)	+ , –	<0.001
BMI and hip	**0.77**(0.73– 0.82)	+ , –	<0.001
Weight, hip and height[b]	**0.78**(0.74–0.83)	+ , – , –	<0.001
BMI, hip and height[c,d]	**0.78**(0.74–0.83)	+ , – , +	<0.001
Men ≥66yrs; n=172			
Weight	0.54(0.48–0.60)	–	>0.20
Height	0.54(0.48–0.60)	–	0.18
Waist	0.56(0.50–0.62)	–	0.060
Hip	**0.72**(0.67–0.76)	–	<0.001
BMI	0.51(0.45–0.57)	–	>0.20
Waist/hip	**0.63**(0.59–0.68)	+	<0.001
Waist and hip	**0.77**(0.72–0.82)	+ , –	<0.001
Weight and hip[c]	**0.82**(0.77–0.86)	+ , –	<0.001
BMI and hip	**0.78**(0.73– 0.83)	+ , –	<0.001
Weight, hip and height[b]	**0.82**(0.77–0.86)	+ , – , –	<0.001
BMI, hip and height[d]	**0.81**(0.76–0.86)	+ , –, +	<0.001
Women ≤65yrs; n=64			
Weight	0.51(0.41–0.62)	-	>0.20
Height	0.54(0.44–0.64)	–	>0.20
Waist	0.52(0.43–0.62)	+	>0.20
Hip	0.57(0.47–0.67)	–	>0.20
BMI	0,54(0.44–0.64)	+	>0.20
Waist/hip	**0.59**(0.49–0.69)	+	0.084
Waist and hip	**0.62**(0.52–0.72)	+ , –	0.011
Weight and hip	**0.66**(0.57–0.76)	+ , –	0.002
BMI and hip[‡]	**0.69**(0.59– 0.78)	+ , –	<0.001
Weight, hip and height[b]	**0.70**(0.61–0.79)	+ , – , –	<0.001
BMI, hip and height[c,d]	**0.71**(0.62–0.80)	+ , – , +	<0.001

Women ≥66yrs; n=100			
Body weight	0.53(0.45–0.61)	+	>0.20
Height	0.52(0.44–0.60)	+	>0.20
Waist	0.56(0.48–0.64)	+	0.15
Hip	0.55(0.47–0.63)	+	>0.20
BMI	0.51(0.44–0.59)	+	>0.20
Waist/hip[c]	**0.65**(0.56–0.71)	+	<0.001
Waist and hip	**0.66**(0.58–0.73)	+ , –	<0.001
Weight and hip	**0.66**(0.59–0.73)	+ , –	<0.001
BMI and hip	**0.62**(0.54– 0.70)	+ , –	0.003
Weight, hip and height[b]	**0.65**(0.57–0.72)	+ , – , –	<0.001
BMI, hip and height[d]	**0.67**(0.60–0.74)	+ , – , +	<0.001

AUROC values larger than or equal 0.60 are in bold.

[a] '+' denotes larger values of the anthropometric measure increases the risk of MI status and '–' denotes larger values decreases the risk.

[b] Best subset of anthropometric measures according to Schwarz Bayesian information criterion taken over all four individual sex/age categories.

[c] Best subset of anthropometric measures according to Schwarz Bayesian information criterion, see Supplementary Table 3.

[d] The '+' direction of the height effect is only apparent. The true direction is negative since it appears in the denominator of BMI.

Supplementary Figure 1. Receiver operating characteristic curves for best anthropometric subset models of myocardial infarction status in women ≤65yrs (left panel) (included variables: hip and BMI) and women ≥66yrs (right panel) (included variable: waist/hip).

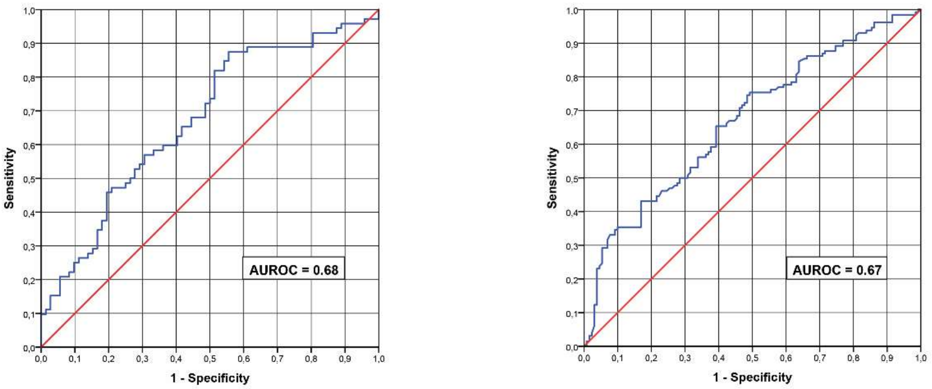

Supplement: Supplementary Materials — The supplementary material section contains 5 tables and 1 figure. Supplementary Table 1 is a more comprehensive version of Table 1. Supplementary Table 2 corresponds to Table 2 but includes only first time MI cases and their matched controls. Supplementary Table 3 contains the numbers for Figure 1. Supplementary Table 4 contains the result of the best subset conditional logistic regression analyses for finding the combination of individual anthropometric measures which best predicted the MI status in the different sex- and age-categories. Supplementary Table 5 corresponds to Table 3 but includes only first time MI cases and their matched controls. Supplementary Figure 1 corresponds to Figure 5 and shows the area under the operating characteristic curves for the best subset of anthropometric measures for women. [file 3839482.f1.pdf]
